# Artificial Intelligence Enabled Fully Automated CMR Function Quantification for Optimized Risk Stratification in Patients Undergoing Transcatheter Aortic Valve Replacement

**DOI:** 10.1155/2022/1368878

**Published:** 2022-04-20

**Authors:** Ruben Evertz, Torben Lange, Sören J. Backhaus, Alexander Schulz, Bo Eric Beuthner, Rodi Topci, Karl Toischer, Miriam Puls, Johannes T. Kowallick, Gerd Hasenfuß, Andreas Schuster

**Affiliations:** ^1^University Medical Center Göttingen (UMG), Department of Cardiology and Pneumology, Göttingen, Germany; ^2^German Center for Cardiovascular Research (DZHK), Partner Site Göttingen, Göttingen, Germany; ^3^University Medical Center Göttingen (UMG), Department of Diagnostic & Interventional Radiology, Göttingen, Germany

## Abstract

**Background:**

Cardiovascular magnetic resonance imaging is considered the reference standard for assessing cardiac morphology and function and has demonstrated prognostic utility in patients undergoing transcatheter aortic valve replacement (TAVR). Novel fully automated analyses may facilitate data analyses but have not yet been compared against conventional manual data acquisition in patients with severe aortic stenosis (AS).

**Methods:**

Fully automated and manual biventricular assessments were performed in 139 AS patients scheduled for TAVR using commercially available software (suiteHEART®, Neosoft; QMass®, Medis Medical Imaging Systems). Volumetric assessment included left ventricular (LV) mass, LV/right ventricular (RV) end-diastolic/end-systolic volume, LV/RV stroke volume, and LV/RV ejection fraction (EF). Results of fully automated and manual analyses were compared. Regression analyses and receiver operator characteristics including area under the curve (AUC) calculation for prediction of the primary study endpoint cardiovascular (CV) death were performed.

**Results:**

Fully automated and manual assessment of LVEF revealed similar prediction of CV mortality in univariable (manual: hazard ratio (HR) 0.970 (95% CI 0.943–0.997) *p*=0.032; automated: HR 0.967 (95% CI 0.939–0.995) *p*=0.022) and multivariable analyses (model 1: (including significant univariable parameters) manual: HR 0.968 (95% CI 0.938–0.999) *p*=0.043; automated: HR 0.963 [95% CI 0.933–0.995] *p*=0.024; model 2: (including CV risk factors) manual: HR 0.962 (95% CI 0.920–0.996) *p*=0.027; automated: HR 0.954 (95% CI 0.920–0.989) *p*=0.011). There were no differences in AUC (LVEF fully automated: 0.686; manual: 0.661; *p*=0.21). Absolute values of LV volumes differed significantly between automated and manual approaches (*p* < 0.001 for all). Fully automated quantification resulted in a time saving of 10 minutes per patient.

**Conclusion:**

Fully automated biventricular volumetric assessments enable efficient and equal risk prediction compared to conventional manual approaches. In addition to significant time saving, this may provide the tools for optimized clinical management and stratification of patients with severe AS undergoing TAVR.

## 1. Introduction

Cardiovascular disease remains the leading cause of death globally. Aortic stenosis (AS) is the most common valvular heart disease and of rising prevalence in the elderly population. Transthoracic echocardiography (TTE) constitutes the standard diagnostic tool to quantify AS using transvalvular gradients and velocities. However, in the absence of a high-gradient situation, the diagnostic work is challenging and includes accurate determination of left ventricular (LV) ejection fraction (EF) and LV stroke volume (SV) to distinguish between different AS subgroups [1]. Furthermore, cardiac function has strong prognostic implications in patients with AS and other structural heart diseases, and therefore, its accurate determination is essential for clinical management and risk prediction [1–5]. Amongst various imaging techniques, cardiovascular magnetic resonance (CMR) imaging is considered a reference methodology with proven superiority over echocardiographical analyses regarding reproducibility and accuracy to detect clinically significant alterations in LV and right ventricular (RV) dimensions and function [6–9]. Recently, novel artificial intelligence (AI)-based deep-learning algorithms were introduced, enabling accurate and fully automated image analyses using convolutional neural networks [10–12]. These AI-based volumetric analyses were already shown to be feasible, reproducible, and of prognostic value in patients with coronary disease and were of high potential for time saving and facilitation of clinical routine [13, 14]. However, similar data in patients with severe AS are currently lacking. Therefore, we sought to investigate fully automated biventricular volumetric analyses using commercially available software solutions in comparison to conventional manual analyses and to study their accuracy in terms of volumetric assessment and prognostic implications in patients with severe AS being scheduled for transcatheter aortic valve replacement (TAVR).

## 2. Methods

### 2.1. Study Population

Patients fulfilling echocardiographic criteria of severe AS according to current guidelines of the European Society of Cardiology and confirmed indications for TAVR without typical contraindications for CMR were able to participate [1, 15, 16]. Between January 2017 and June 2021, a total of 146 patients were prospectively enrolled and agreed to an additional CMR before undergoing TAVR as part of an interdisciplinary research project on aortic valve stenosis [17]. The local ethics committee approved the study, and written informed consent was obtained from all patients. The study was conducted according to the principles of the Helsinki Declaration.

### 2.2. Clinical End Points

Death from cardiovascular (CV) reason according to the VARC-3 definition was defined as the clinical end point of this study [18].

### 2.3. CMR Analyses

**Figure 1:**
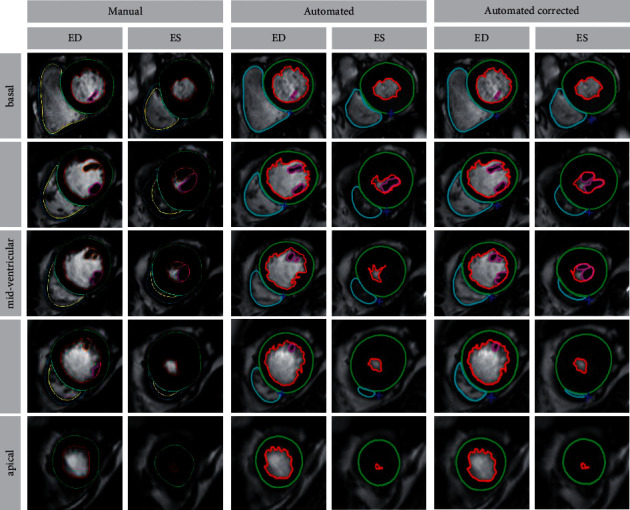
Manual, automated, and automated-corrected biventricular volumetric analyses. Overview of a tracked short-axis stack from the base to apex in end-diastole (ED) and end-systole (ES) using manual and automated analysis software.

CMR imaging was performed on a 3 Tesla MR scanner (MAGNETOM Skyra, Siemens Healthcare, Erlangen, Germany) using a 32-channel surface coil. The standardized scanning protocol has been reported elsewhere and included long- and short-axis (SAX) steady-state free precession images (repetition time, 3.2 ms; echo time, 1.2 ms; flip angle. 60°; slice thickness 8 mm) [19]. An experienced investigator performed manual volumetric analyses in short-axis orientation using a dedicated postprocessing software (QMass®, Version 3.2.36.4, Medis Medical Imaging Systems, Leiden, Netherlands) according to current clinical recommendations including papillary muscles within the myocardium [20]. For automated volumetric analyses, commercially available AI software provided by Neosoft (suiteHEART, Version 5.0.0, Neosoft, Pewaukee, Wisconsin, USA) was used. In a first step, after uploading the complete dataset of all patients, fully automated analyses were performed overnight without any further postprocessing user interaction. Afterwards, all automatically traced endocardial and epicardial borders were reviewed visually and adapted in case of insufficient border delineation. Furthermore, the time needed for visual border validation and, if required, contour correction was recorded. Volumetric analyses included LV mass, LV and RV end-diastolic/-systolic (EDV/ESV) volumes, stroke volume (SV), and LV and RV EF (Figure 1).

### 2.4. Statistical Analysis

Statistical analysis was performed using IBM SPSS Statistics version 27 for Windows (International Business Machines Corporation (IBM® Corp.), Armonk, New York, United States of America) and Microsoft Excel 2016 (Microsoft Corporation, Redmond, Washington, USA). Normal distribution for continuous data was tested using the Shapiro–Wilk test. Data were compared using the Mann–Whitney *U* or Student's *t*-test as appropriate and expressed as median and interquartile range. Intergroup comparison of categorical variables was performed using the *χ*^2^ test, and data were presented as absolute numbers and percentages. Dependent variables were tested using the Wilcoxon signed rank test or Student's *t*-test for paired samples as appropriate. Assessment of the manual and automated analyses agreement was performed first by calculation of the intraclass correlation coefficients (ICC), which was scored as excellent (>0.74), good (0.6–0.74), fair (0.4–0.59), and poor (<0.4), second by Bland–Altman analysis (mean difference between measurements with 95% confidence interval (CI)), and third by the coefficient of variation (COV) [21, 22]. COV was defined as the standard deviation of the differences divided by the mean [23]. Univariable calculations were used to identify determinants of the predefined end point and included in multivariable calculations if *p* < 0.05 (model 1). In a second model, classical CV risk factors were additionally included (age, hypertension, diabetes mellitus, dyslipidaemia, and coronary heart disease). Results of regression analyses were expressed as hazard ratio (HR) with corresponding 95% confidence intervals (CIs). To assess the additional predictive value of automatically generated volumetric parameters, receiver operator characteristics (ROC) were implemented. For both manual and automatic measurements, the area under the curve (AUC) for predicting the endpoint was calculated and compared using the nonparametric approach by DeLong et al. [24].

## 3. Results

### 3.1. Study Population

While the initial study population consisted of 146 patients, the final cohort after withdrawal was 142 patients. These consisted of 71 patients (50.0%) with normal ejection fraction high gradient (NEFHG) AS; 19 patients (13.4%) with low ejection fraction high gradient (LEFHG) AS; 21 patients (14.8%) with low ejection fraction low gradient (LEFLG) AS; and 31 patients (21.8%) with paradoxical low flow low gradient (PLFLG) AS. Mean age of the study population was 78 ± 6 years with age ranging from 59 to 90 years. The majority of patients (62%, *n* = 88) were male. Predominant comorbidities were hypertension (85.9%) followed by coronary artery disease (65.5%), atrial fibrillation (32.4%), stroke/transient ischemic attack (TIA) (12.7%), and chronic obstructive pulmonary disease (COPD) (9.9%). CV death occurred in 12.0% of patients. There were no differences between survivors and deceased patients in regard to age, sex, and comorbidities. However, deceased patients' BMI was slightly higher compared to survivors (*p*=0.017). Details are displayed in Table 1.

### 3.2. Automated and Manual Assessment of the Volumetric Parameters

Of the finally included 142 patients, 142 (100%) patients were analyzed manually and 139 (97.9%) patients automatically, because the fully automated analysis did not work. Therefore, further analyses were performed with the remaining 139 patients.

Differences between manual and automated biventricular segmentation are presented in Table 2. LV mass was estimated higher and LV volumes lower using automated analyses compared to manual analyses (LV mass index (g/m^2^) automated vs. manual: 88.0 [75.0–111.0] vs. 83.3 [69.4–102.8]; LVEDV index (ml/m^2^) automated vs. manual: 71.3 [60.0–88.8] vs. 78.3 [63.3–97.3]; LVESV index (ml/m^2^) automated vs. manual: 27.7 [16.0–45.6] vs. 31.1 [17.9–44.9] all *p* < 0.001). The opposite was true for RV volumes with statistically significant differences for RVEDV (RVEDV index (ml/m^2^) automated vs. manual: 69.4 [58.4–83.0] vs. 67.3 [56.9–80.8] *p* < 0.001; RVESV index (ml/m^2^) automated vs. manual: 31.7 [22.7–39.9] vs. 31.4 [23.1–44.4] *p*=0.07). RVEF was higher using automated analyses, but not LVEF (RVEF (%) automated vs. manual: 55.0 [9.0–61.0] vs. 53.6 [44.2–59.7] *p*=0.01; LVEF (%) automated vs. manual: 62.0 [46.0–73.0] vs. 60.3 [45.9–73.4] *p*=0.889). Similar findings were observed in AS subgroups and are presented in the online data supplement (Tables S1–S4).

**Figure 2:**
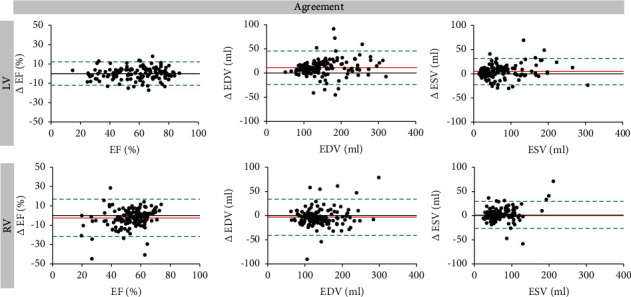
Bland–Altman plots for agreement of manual and automated biventricular volumes. LV: left ventricular; RV: right ventricular; EF: ejection fraction; EDV: end-diastolic volume; ESV: end-systolic volume.

Table 3 illustrates the agreement of fully automated and manual analyses including bias with 95% limits of agreement (LOA), ICC, and COV. In addition, Bland–Altman plots are presented in Figure 2. Overall, for both the LV and RV measurements, high agreement was found between manual and automated analyses. However, LV parameters showed better agreement than RV parameters for LVEF (bias: 0; 95% LOA: −12.1 to 12.1; ICC 0.964; COV: 10.5), LVEDV (bias: 11.13; 95% LOA: −23, 5 to 45.8; ICC 0.978; COV: 11.2), and LVESV (bias 4.63; 95% LOA: −22.6 to 31.9; ICC 0.983; COV: 19.5) as compared to RVEF (bias: −2.44; 95% LOA: −21.7 to 16.9; ICC 0.804; COV: 18.6), RVEDV (bias: −3.44; 95% LOA: −40.7 to 33.8; ICC 0.954; COV: 13.6), and RVESV (bias: 1.37; 95% LOA: −26.6 to 29.4; ICC 0.955; COV: 21.0). Data for corresponding subgroup analyses are presented in the online data supplement (Tables S5–S8).

Manual postprocessing volumetric analyses took on average 13 minutes by an experienced operator. In contrast, using fully automated software took on average 45 seconds for volumetric analyses. The consequent operator review of the correct contour detection took 60 seconds on average. Correction of the contours took another 60 seconds on average if needed. In 22 patients (15.8%), minor manual corrections of the myocardial borders mainly in the most basal or apical slices were performed resulting in a better agreement with manual analyses (Table 4).

### 3.3. Prognostic Value of Automated and Manual Assessments

The mean follow-up period was 760 ± 439 days. During this period a total of 27 patients (19.4%) died, whereas in 17 cases (12.2%), a cardiovascular death occurred. Cox regression univariable modelling revealed that BMI (HR 1.090 (95% CI 1.001–1.187) *p*=0.048) and the presence of COPD (HR 3.090 (95% CI 1.005–9.501) *p*=0.048) were associated with increased CV mortality. Regarding volumetric parameters, both manual and automated LVEF were associated with the occurrence of CV death (manual: HR 0.970 (95% CI 0.943–0.997) *p*=0.032; automated: HR 0.967 (95% CI 0.939–0.995) *p*=0.022). LVEF, derived manually or fully automatically, remained a significant predictor of CV death on multivariable modelling including significant univariable parameters (manual: HR 0.968 (95% CI 0.938–0.999) *p*=0.043; automated: HR 0.963 (95% CI 0.933–0.995) *p*=0.024]. In a second model, classical CV risk factors were additionally included (age, hypertension, diabetes mellitus, dyslipidaemia, coronary heart disease). LVEF remained a significant predictor of CV death (manual: HR 0.962 (95% CI 0.920–0.996) *p*=0.027; automated: HR 0.954 (95% CI 0.920–0.989) *p*=0.011). In either model, BMI was also an independently significant risk predictor. A detailed overview is given in Table 5.

There were no significant differences seen between fully automated, automated corrected, and manual LVEFs on AUC comparison (fully automated: AUC 0.686; automated corrected: AUC: 0.671; manual: AUC 0.661; fully automated vs. automated corrected: *p*=0.115, fully automated vs. manual: *p*=0.214, automated corrected vs. manual: *p*=0.545).

## 4. Discussion

To our knowledge, this is the first study investigating the applicability of an AI-based fully automated biventricular volumetric and functional analysis with demonstrated clinical utility and predictive value for optimized risk stratification in patients with severe AS. The following findings are notable: Firstly, fully automatically derived CMR-based LVEF has a similar significant association with mortality compared to conventional analyses with the advantage of a substantial time saving. Secondly, automatically calculated results seem sufficient for risk prediction without a mandatory user interaction by a CMR imaging specialist. Thirdly, AI-based CMR postprocessing software facilitates the use and widens the applicability of CMR imaging with potential fast and easy integration into clinical routine.

The rising incidence of valvular heart diseases is inevitably associated with an increased need for economical and accurate diagnostic procedures. Especially CMR imaging plays a key role amongst noninvasive imaging techniques due to its comprehensive myocardial analysis tools. However, its postprocessing routine is still laborious and time consuming [8]. Recently, automated postprocessing software solutions based on deep-learning algorithms have been developed and are already commercially available with proven clinical utility [11, 25, 26].

AI software has been already applied in various cardiovascular diseases and shown to offer similar or even improved risk stratification compared to manual approaches [27]. Applications are wide ranging and demonstrate that a patient-centred individual approach, for example, using machine learning multiprotein risk models, allows a better detection of future events than currently used clinical risk scores [28]. Recently, the field of applications has also been extended to optimized screening and diagnosis procedures including subtle ECG alterations in patients with AS [29, 30]. Amongst the parameters for clinical decision making, especially the LVEF has a pivotal role for optimized patient management with important prognostic implications that were proven in various different studies comprising common CV diseases like acute myocardial infarction or heart failure [2, 31, 32]. Furthermore, clinical decisions like the indication for the implantation of an implantable cardioverter defibrillator are based on the LVEF [2]. In addition, in patients with severe AS scheduled for aortic valve replacement, data have shown an important association between mortality and LVEF [33, 34]. In line with these findings, our results showed significant associations of the LVEF with CV mortality independently of whether a fully automated or conventional analysis approach was used. Therefore, applying fully automated volumetric analyses in patients with severe AS is feasible and offers an attractive alternative postprocessing approach compared to manual segmentation with equal prognostic implications. Although the LVEF might not be the one complete parameter to describe the prognosis of all AS patients, for example, aortic valve calcification or global longitudinal strain measurements might be the more important parameters for optimized prognosis evaluation in different subgroups of AS, the LVEF and the SV have important roles to define respective AS subgroups and, therefore, their accurate assessment has a crucial role in clinical routine [35, 36]. Furthermore, our results are similar to the predictive value of LVEF in a large cohort of patients with acute myocardial infarction and therefore confirm a certain predictive value of this parameter in patients with AS [13].

Besides an equal risk prediction, there were numerical differences between fully automatically assessed volumetric parameters compared to manual segmentation by an experienced CMR operator in our study. Fully automated measurements resulted in larger LV mass and smaller LV volumes; however, the LVEF showed no statistically significant difference. These results are contrary to previous studies applying AI-based fully automated quantification that documented smaller LV mass but larger volumes [13, 14]. These differences might be caused by a slightly different volumetric approach excluding the papillary muscles and trabecular endocardial tissue in the aforementioned studies. Since an exact delineation of trabecular tissue using manual analysis software is tedious and partially limited due to automated smoothing of the analysis software, the automated approach might provide a more exact representation of myocardial volumetric relations. Nevertheless, in line with the aforementioned studies, the documented agreement of volumetric parameters was excellent in our study with LVEF having the lowest bias. In terms of biventricular agreement, LV measurements were better than RV segmentations, which might be explainable by the more complex anatomy of the RV which has also been described in previous CMR studies [14, 37].

Even though fully automated analysis was successful in the majority of the study population (97.9%), a visual review of the automated contours and their adaption, if necessary, by the CMR operator was performed in our study and resulted in an improved agreement of volumetric parameters. However, the corrections did not enable an improved risk stratification, and therefore, a direct clinical use of the parameters without a categorically needed review of the delineations could be envisaged. Although one might consider to omit visual review of the contours as a consequence, individual level CIs of −12.1 to 12.1 for LVEF and even wider ranges for LV volumes underline the importance of a visual review and corrections in case of insufficient border delineation. As previously described, a relevant proportion of patients required manual border adjustments especially in basal and apical slices which are the most challenging areas of myocardial volumetric analyses bringing current automated software solutions to their delineation limits [13, 38].

However, the decisive advantage of AI-based software in the field of CMR postprocessing is a remarkable saving of time, which is underlined by the results of our study. Compared to manual analysis, the AI-based software provided about 10 saved minutes for volumetric assessments on a per-patient basis. The time saving use of fully automated software can be even increased by using it “on-the-fly” during imaging acquisition or overnight. This does not only result in a more efficient postprocessing practice during clinical routine but also in facilitated analyses of large patient cohorts and, consequently, might even be accompanied by lower costs of CMR imaging procedures.

In addition to the time-saving aspect, AI solutions offer more user-independent measurements and can improve comparability of parameters in serial examinations or between CMR core laboratories of different hospitals. The excellent intra-observer and interobserver reproducibility for fully automated volumetric assessment that exceeds the reproducibility of manual assessments has been described previously by Backhaus et al. [14]. With a more widespread availability of MRI scanners and increasing incidences of patients with AS and consequently rising numbers of interventional valve replacement procedures, AI-based software therefore constitutes a key tool for accurate and efficient volumetric assessment in clinical routine even for nonimaging specialists.

### 4.1. Study Limitations

Some limitations need to be addressed. Firstly, due to typical CMR contraindications only selected patients were able to participate in this study. Secondly, only patients considered stable and being able to lie in a supine position were included. Both contraindications and the ability to undergo CMR scanning might have led to a selection bias and resulting in lower event rates by excluding potentially sicker patients. However, these limitations apply to both analysis techniques and therefore do not limit the validity of the analysis. Thirdly, detailed information of the AI-based algorithm is not disclosed by the manufacturer and therefore cannot be described in more detail. Thirdly, the fully automated software does not offer RV mass quantification yet, and consequently, this parameter was not analyzed in our study. Fourthly, a total of three patients (2.1%) could not be analyzed using the automated algorithm, which needs to be considered especially when studying dyspneic patients such as AS patients. Finally, we have observed small numerical differences between fully automated and manual volumetric assessments. Consequently, on an individual patient level final contours and results should always be approved or corrected by a responsible physician to also allow comparability between repeated scans, e.g., before and after TAVR.

## 5. Conclusion

Fully automated assessment of biventricular volumes and function is feasible and enables similar risk prediction compared to a conventional manual approach in patients with severe aortic stenosis scheduled for TAVR. Agreement between manual and fully automated analyses is excellent, and manual correction of border delineation does not lead to an improved risk prediction. Due to its accuracy and immense time-saving nature, application of AI software enables a more widespread user-independent risk stratification and may facilitate easy implementation of CMR imaging in clinical routine prior to TAVR. Further studies are needed to validate these findings to fully establish this technique in clinical routine.

## Figures and Tables

**Table 1:** Baseline characteristics

Variable	All patients (*n* = 142)	Survivors (*n* = 125)	CV deceased (*n* = 17)	*p* value
Age (Y)	80 (74–83)	79 (74–82)	82 (78.5–84)	0.069
Sex (male)	88 (62.0%)	77 (61.6%)	11 (64.7%)	0.805
BMI (kg/m^2^)	27.5 (24.6–30.7)	27.0 (24.4–30.2)	30.8 (26.9–33.5)	0.014
Comorbidities				
Hypertension	122 (85.9%)	107 (85.6%)	15 (88.2%)	1.000
Diabetes mellitus	46 (32.4%)	37 (29.6%)	9 (52.9%)	0.054
Dyslipidaemia	97 (68.3%)	86 (68.8%)	11 (64.7%)	0.866
Coronary artery disease	93 (65.5%)	80 (64.0%)	13 (76.5%)	0.310
Atrial fibrillation	46 (32.4%)	38 (30.4%)	8 (47.1%)	0.168
Stroke/TIA	18 (12.7%)	16 (12.8%)	2 (11.8%)	0.926
COPD	14 (9.9%)	10 (8.0%)	4 (23.5%)	0.066

Data are expressed as median (interquartile range), numbers, and percentage. Comparison of survivors and deceased was performed. Continuous parameters were tested for normal distribution using the Shapiro–Wilk test and compared using the Mann–Whitney *U* test or *t*-test as appropriate. Categorical parameters were tested using the chi -square test. BMI: body mass index; TIA: transient ischemic attack; COPD: chronic obstructive pulmonary disease.

**Table 2:** Biventricular volumes based on CMR measurements.

Left ventricle	Automated (uncorrected)	Manual	*p* value

LV mass (g)	170.1 (139.1–213.9)	161 (132.0–199.2)	<0.001
LV mass index (g/m^2^)	88.0 (75.0–111.0)	83.3 (69.4–102.8)	<0.001
LV EDV index (ml/m^2^)	71.3 (60.0–88.8)	78.3 (63.3–97.3)	<0.001
LV ESV index (ml/m^2^)	27.7 (16.0–45.6)	31.1 (17.9–44.9)	<0.001
LV SV index (ml/m^2^)	42.8 (35.3–49.3)	45.5 (36.7–53.9)	<0.001
LVEF (%)	62.0 (46.0–73.0)	60.3 (45.9–73.4)	0.889

Right ventricle	Automated (uncorrected)	Manual	*p* value
RV EDV index (ml/m^2^)	69.4 (58.4–83.0)	67.3 (56.9–80.8)	<0.001
RV ESV index (ml/m^2^)	31.7 (22.7–39.9)	31.4 (23.1–44.4)	0.07
RV SV index (ml/m^2^)	38.6 (31.4–45.0)	35.2 (28.8–43.4)	<0.001
RVEF (%)	55.0 (49.0–61.0)	53.6 (44.2–59.7)	0.01

Continuous data were compared using the Wilcoxon signed rank test and are expressed as median (interquartile range). EDV: end-diastolic volume; ESV: end-systolic volume; LV: left ventricular; LVEF: left ventricular ejection fraction; RV: right ventricular; RVEF: right ventricular ejection fraction; SV: stroke volume.

**Table 3:** Agreement between manual and automated uncorrected analyses.

Left ventricle	Bias	95% LOA	ICC (95% CI)	COV (%)

LV mass (g)	−10.08	−84.2 to 64.1	0.890 (0.846–0.921)	21.5
LV EDV (ml)	11.13	−23.5 to 45.8	0.978 (0.969–0.984)	11.2
LV ESV (ml)	4.63	−22.6 to 31.9	0.983 (0.977–0.988)	19.5
LV SV (ml)	6.69	−17.6 to 31.0	0.935 (0.909–0.954)	14.4
LVEF (%)	0	−12.1 to 12.1	0.964 (0.950–0.975)	10.5

Right ventricle	Bias	95% LOA	ICC (95% IC)	COV (%)
RV EDV (ml)	−3.44	−40.7 to 33.8	0.954 (0.936–0.967)	13.6
RV ESV (ml)	1.37	−26.6 to 29.4	0.955 (0.938–0.968)	21.0
RV SV (ml)	−4.26	−37.1 to 28.6	0.832 (0.765–0.880)	23.3
RVEF (%)	−2.44	−21.7 to 16.9	0.804 (0.725–0.860)	18.6

EDV: end-diastolic volume; ESV: end-systolic volume; LV: left ventricular; LVEF: left ventricular ejection fraction; RV: right ventricular; RVEF: right ventricular ejection fraction; SV: stroke volume.

**Table 4:** Agreement between manual and automated corrected analyses.

Left ventricle	Bias	95% LOA	ICC (95% CI)	COV (%)

LV mass (g)	−9.91	−83.6 to 63.8	0.891 (0.848–0.922)	21.4
LV EDV (ml)	11.47	−22.5 to 45.4	0.979 (0.971–0.985)	11.0
LV ESV (ml)	4.77	−20.8 to 30.3	0.985 (0.979–0.989)	18.4
LV SV (ml)	6.89	−15.8 to 29.6	0.944 (0.921–0.960)	13.5
LVEF (%)	0.03	−10.2 to 10.2	0.975 (0.965–0.982)	8.8

Right ventricle	Bias	95% LOA	ICC (95% IC)	COV (%)
RV EDV (ml)	−3.48	−40.7 to 33.7	0.954 (0.936–0.967)	13.6
RV ESV (ml)	1.44	−25.5 to 28.4	0.958 (0.942–0.970)	20.3
RV SV (ml)	−4.35	−36.3 to 27.6	0.841 (0.778–0.886)	22.7
RVEF (%)	−2.47	−21.4 to 16.5	0.810 (0.735–0.864)	18.3

EDV: end-diastolic volume; ESV: end-systolic volume; LV: left ventricular; LVEF: left ventricular ejection fraction; RV: right ventricular; RVEF: right ventricular ejection fraction; SV: stroke volume.

**Table 5:** Univariable and multivariable cox regression analyses including LVEF for prediction of CV mortality.

Variable	Hazard ratio (95% CI)	*p*value
Univariable models		
Age (Y)	1.074 (0.978–1.178)	0.135
Sex (male)	1.195 (0.438–3.261)	0.727
BMI (kg/m^2^)	1.090 (1.001–1.187)	**0.048**
Hypertension (present)	1.054 (0.240–4.623)	0.944
Diabetes mellitus (present)	2.196 (0.846–5.700)	0.106
Dyslipidaemia (present)	0.953 (0.347–2.618)	0.925
Coronary artery disease (present)	1.888 (0.614–5.811)	0.268
Atrial fibrillation (present)	2.372 (0.907–6.198)	0.078
Stroke/TIA (present)	0.816 (0.186–3.573)	0.787
COPD (present)	3.090 (1.005–9.501)	**0.049**
Automated LVEF (%)	0.967 (0.939–0.995)	**0.022**
LVEF (%)	0.970 (0.943–0.997)	**0.032**
Automated LV SVI (ml/m^2^)	0.996 (0.952–1.042)	0.859
LV SVI (ml/m^2^)	0.999 (0.961–1.039)	0.965
Multivariable models		
Model 1a		
Automated LVEF (%)	0.963 (0.933–0.995)	**0.024**
BMI (kg/m^2^)	1.130 (1.029–1.241)	**0.011**
COPD (present)	2.277 (0.691–7.507)	0.451
Model 1b		
LVEF (%)	0.968 (0.938–0.999)	**0.043**
BMI (kg/m^2^)	1.126 (1.025–1.237)	**0.013**
COPD (present)	2.400 (0.718–8.014)	0.155
Model 2a		
Automated LVEF (%)	0.954 (0.920–0.989)	**0.011**
BMI (kg/m^2^)	1.162 (1.024–1.320)	**0.020**
COPD (present)	1.718 (0.414–7.123)	0.456
Age (Y)	2.231 (0.742–6.704)	0.153
Diabetes mellitus (present)	2.231 (0.742–6.704)	0.153
Hypertension (present)	2.128 (0.397–11.417)	0.378
Dyslipidaemia (present)	0.662 (0.209–2.090)	0.482
Coronary artery disease (present)	1.363 (0.391–4.747)	0.627
Model 2b		
LVEF (%)	0.962 (0.929–0.996)	**0.027**
BMI (kg/m^2^)	1.139 (1.014–1.280)	**0.028**
COPD (present)	2.000 (0.494–8.102)	0.331
Age (Y)	1.113 (0.345–4.406)	0.055
Diabetes mellitus (present)	2.385 (0.798–7.121)	0.120
Hypertension (present)	1.846 (0.356–9.578)	0.465
Dyslipidaemia (present)	0.655 (0.209–2.055)	0.468
Coronary artery disease (present)	1.233 (0.345–4.406)	0.747

BMI: body mass Index; TIA: transient ischemic attack; COPD: chronic obstructive pulmonary disease; LVEF: left ventricular ejection fraction; LV SVI: left ventricular stroke volume index

## Data Availability

Regarding data availability, we confirm that all relevant data are included within the paper and all data underlying the findings are fully available without restriction and can be accessed at the University Medical Centre Goettingen by researchers who meet the criteria for access to confidential data.

## Supplementary Materials

Supplementary Materials Table S1: biventricular volumes based on CMR measurements in patients with normal ejection fraction high gradient aortic stenosis (*n* = 68). Table S2: biventricular volumes based on CMR measurements in patients with low ejection fraction high gradient aortic stenosis (*n* = 19). Table S3: biventricular volumes based on CMR measurements in patients with low ejection fraction low gradient aortic stenosis (*n* = 21). Table S4: biventricular volumes based on CMR measurements in patients with paradoxical low flow low gradient aortic stenosis (*n* = 31). Table S5: agreement between manual and automated uncorrected analyses in patients with normal ejection fraction high gradient aortic stenosis (*n* = 68). Table S6: agreement between manual and automated uncorrected analyses in patients with low ejection fraction high gradient aortic stenosis (*n* = 19). Table S7: agreement between manual and automated uncorrected analyses in patients with low ejection fraction low gradient aortic stenosis (*n* = 21). Table S8: agreement between manual and automated uncorrected analyses in in patients with paradoxical low flow low gradient aortic stenosis (*n* = 31).
