# Supplementary material for: Artificial Intelligence Enabled Fully Automated CMR Function Quantification for Optimized Risk Stratification in Patients Undergoing Transcatheter Aortic Valve Replacement
Source: J Interv Cardiol. 2022 Apr 20;2022:1368878. doi: 10.1155/2022/1368878 (PMC9046000; doi:10.1155/2022/1368878)
Supplement: Supplementary Materials — Table S1: biventricular volumes based on CMR measurements in patients with normal ejection fraction high gradient aortic stenosis (n = 68). Table S2: biventricular volumes based on CMR measurements in patients with low ejection fraction high gradient aortic stenosis (n = 19). Table S3: biventricular volumes based on CMR measurements in patients with low ejection fraction low gradient aortic stenosis (n = 21). Table S4: biventricular volumes based on CMR measurements in patients with paradoxical low flow low gradient aortic stenosis (n = 31). Table S5: agreement between manual and automated uncorrected analyses in patients with normal ejection fraction high gradient aortic stenosis (n = 68). Table S6: agreement between manual and automated uncorrected analyses in patients with low ejection fraction high gradient aortic stenosis (n = 19). Table S7: agreement between manual and automated uncorrected analyses in patients with low ejection fraction low gradient aortic stenosis (n = 21). Table S8: agreement between manual and automated uncorrected analyses in in patients with paradoxical low flow low gradient aortic stenosis (n = 31). [file 1368878.f1.docx]

Supplement

| **Left ventricle** | **Automated (uncorrected)** | **Manual** | **p-value** |
| --- | --- | --- | --- |
| LV mass [g] | 173.1 (145.0 – 211.5) | 162.2 (134.9 – 188.2) | **< 0.001** |
| LV mass index [g/m²] | 89.0 (74.8 – 108.8) | 84.4 (66.2 – 98.8) | **< 0.001** |
| LV EDV index [ml/m²] | 67.9 (56.5 – 79.7) | 72.6 (61.1 – 86.4) | **< 0.001** |
| LV ESV index [ml/m²] | 18.8 (12.8 – 29.5) | 19.2 (14.2 – 32.1) | **0.01** |
| LV SV index [ml/m²] | 45.7 (40.5 – 52.0) | 51.4 (41.1 – 58.1) | **< 0.001** |
| LVEF [%] | 72.0 (63.0 – 78.8) | 70.9 (60.3 – 77.6) | 0.753 |
| **Right ventricle** | **Automated (uncorrected)** | **Manual** | **p-value** |
| RV EDV index [ml/²] | 66.9 (58.0 – 79.9) | 65.4 (56.7 – 79.6) | 0.089 |
| RV ESV index [ml/m²] | 28.8 (22.1 – 33.6) | 27.6 (23.0 – 35.7) | 0.638 |
| RV SV index [ml/m²] | 40.5 (33.4 – 46.6) | 37.8 (32.1 – 46.1) | 0.082 |
| RVEF [%] | 58.5 (54.0 – 63.8) | 56.1 (51.4 – 61.7) | 0.109 |

**Table S1** Biventricular volumes based on CMR measurements in patients with normal ejection fraction high gradient Aortic stenosis (n = 68). Continuous data were compared using Wilcoxon singed rank test or Student´s t test as appropriate and are expressed as median (interquartile range). EDV: end-diastolic volume; ESV: end-systolic volume; LV: left ventricular; LVEF: left ventricular ejection fraction; RV: right ventricular; RVEF: right ventricular ejection fraction; SV: stroke volume.

| **Left ventricle** | **Automated (uncorrected)** | **Manual** | **p-value** |
| --- | --- | --- | --- |
| LV mass [g] | 213.0 (178.7 – 244.8) | 205.7 (177.4 – 232.6) | **< 0.022** |
| LV mass index [g/m²] | 113.0 (98.0 – 137.0) | 108.0 (99.0 – 124.0) | **< 0.016** |
| LV EDV index [ml/²] | 106.9 (86.5 – 123.8) | 106.1 (92.6 – 136.5) | **0.027** |
| LV ESV index [ml/m²] | 62.7 (46.2 – 76.8) | 67.0 (45.7 – 87.0) | 0.059 |
| LV SV index [ml/m²] | 45.5 (40.4 – 52.0) | 50.0 (37.3 – 55.1) | **0.039** |
| LVEF [%] | 45.0 (37.0 – 52.0) | 42.1 (37.0 – 50.2) | 0.880 |
| **Right ventricle** | **Automated (uncorrected)** | **Manual** | **p-value** |
| RV EDV index [ml/²] | 78.5 (61.8 – 95.1) | 72.5 (59.4 – 90.7) | **0.002** |
| RV ESV index [ml/m²] | 34.1 (19.8 – 50.2) | 32.0 (21.4 – 55.6) | 0.438 |
| RV SV index [ml/m²] | 40.9 (33.4 – 46.2) | 36.1 (29.7 – 42.5) | **0.046** |
| RVEF [%] | 53.0 (48.0 – 67.0) | 52.1 (37.9 – 67.3) | 0.122 |

**Table S2** Biventricular volumes based on CMR measurements in patients with low ejection fraction high gradient Aortic stenosis (n = 19). Continuous data were compared using Wilcoxon singed rank test or Student´s t test as appropriate and are expressed as median (interquartile range). EDV: end-diastolic volume; ESV: end-systolic volume; LV: left ventricular; LVEF: left ventricular ejection fraction; RV: right ventricular; RVEF: right ventricular ejection fraction; SV: stroke volume.

| **Left ventricle** | **Automated (uncorrected)** | **Manual** | **p-value** |
| --- | --- | --- | --- |
| LV mass [g] | 201.8 (161.0 – 244.4) | 181.5 (152.7 – 210.1) | **0.032** |
| LV mass index [g/m²] | 97.0 (80.5 – 131.0) | 88.8 (76.2 – 109.5) | **0.036** |
| LV EDV index [ml/²] | 105.8 (72.8 – 135.2) | 109.4 (83.5 – 133.6) | **< 0.001** |
| LV ESV index [ml/m²] | 68.0 (39.6 – 87.6) | 79.0 (41.1 – 89.9) | **0.023** |
| LV SV index [ml/m²] | 37.3 (32.7 – 44.4) | 40.9 (33.6 – 48.0) | 0.196 |
| LVEF [%] | 36.0 (30.5 – 48.5) | 38.0 (28.0 – 46.9) | 0.901 |
| **Right ventricle** | **Automated (uncorrected)** | **Manual** | **p-value** |
| RV EDV index [ml/²] | 80.6 (65.0 – 95.7) | 71.7 (59.5 – 89.5) | 0.222 |
| RV ESV index [ml/m²] | 39.9 (34.4 – 63.0) | 43.2 (33.1 – 58.4) | **0.023** |
| RV SV index [ml/m²] | 38.0 (28.7 – 41.7) | 31.7 (24.1 – 35.9) | 0.053 |
| RVEF [%] | 49.0 (35.0 – 53.5) | 40.3 (29.5 – 51.8) | 0.053 |

**Table S3** Biventricular volumes based on CMR measurements in patients with low ejection fraction low gradient Aortic stenosis (n = 21). Continuous data were compared using Wilcoxon singed rank test or Student´s t test as appropriate and are expressed as median (interquartile range). EDV: end-diastolic volume; ESV: end-systolic volume; LV: left ventricular; LVEF: left ventricular ejection fraction; RV: right ventricular; RVEF: right ventricular ejection fraction; SV: stroke volume.

| **Left ventricle** | **Automated (uncorrected)** | **Manual** | **p-value** |
| --- | --- | --- | --- |
| LV mass [g] | 133.6 (114.7 – 163.2) | 127.2 (103.4 – 161.9) | **0.002** |
| LV mass index [g/m²] | 75.0 (62.0 – 85.0) | 69.4 (58.4 – 79.2) | **0.001** |
| LV EDV index [ml/²] | 60.9 (53.4 – 71.3) | 67.3 (57.1 – 76.8) | **< 0.001** |
| LV ESV index [ml/m²] | 23.9 (18.4 – 31.7) | 26.6 (17.7 – 37.7) | 0.092 |
| LV SV index [ml/m²] | 37.1 (30.8 – 42.5) | 38.9 (32.9 – 44.5) | **0.010** |
| LVEF [%] | 62.0 (49.0 – 68.0) | 66.1 (49.6 – 68.4) | 0.754 |
| **Right ventricle** | **Automated (uncorrected)** | **Manual** | **p-value** |
| RV EDV index [ml/²] | 66.1 (57.9 – 80.6) | 60.9 (55.9 – 77.6) | 0.240 |
| RV ESV index [ml/m²] | 32.6 (25.0 – 43.8) | 32.4 (23.0 – 44.4) | 0.337 |
| RV SV index [ml/m²] | 34.0 (27.1 – 42.8) | 33.0 (26.0 – 40.8) | 0.189 |
| RVEF [%] | 53.0 (46.0 – 59.0) | 49.3 (43.0 – 59.2) | 0.659 |

**Table S4** Biventricular volumes based on CMR measurements in patients with paradoxical low flow low gradient Aortic stenosis (n = 31). Continuous data were compared using Wilcoxon singed rank test or Student´s t test as appropriate and are expressed as median (interquartile range). EDV: end-diastolic volume; ESV: end-systolic volume; LV: left ventricular; LVEF: left ventricular ejection fraction; RV: right ventricular; RVEF: right ventricular ejection fraction; SV: stroke volume.

| **Left ventricle** | **Bias** | **95 % LOA** | **ICC (95 % IC)** | **COV (%)** |
| --- | --- | --- | --- | --- |
| LV mass [g] | -7.97 | -105.4 to 89.5 | 0.818 (0.705 – 0.888) | 28.1 |
| LV EDV [ml] | 11.13 | -19.1 to 41.4 | 0.960 (0.936 – 0.976) | 11.1 |
| LV ESV [ml] | 2.71 | -17.8 to 23.2 | 0.959 (0.934 – 0.975) | 23.2 |
| LV SV [ml] | 8.92 | -13.7 to 31.6 | 0.935 (0.895 – 0.960) | 12.3 |
| LVEF [%] | 0.17 | -11.9 to 12.2 | 0.915 (0.862 – 0.948) | 8.9 |
| **Right ventricle** | **Bias** | **95 % LOA** | **ICC (95 % IC)** | **COV (%)** |
| RV EDV [ml] | -0.90 | -35.6 to 33.8 | 0.934 (0.893 – 0.959) | 13.1 |
| RV ESV [ml] | 1.42 | -18.6 to 21.4 | 0.940 (0.902 – 0.963) | 17.5 |
| RV SV [ml] | -2.74 | -32.3 to 26.8 | 0.830 (0.725 – 0.895) | 19.7 |
| RVEF [%] | -1.52 | -16.7 to 13.6 | 0.720 (0.545 – 0.827) | 13.5 |

**Table S5** Agreement between manual and automated uncorrected analyses in patients with normal ejection fraction high gradient Aortic stenosis (n = 68). EDV: end-diastolic volume; ESV: end-systolic volume; LV: left ventricular; LVEF: left ventricular ejection fraction; RV: right ventricular; RVEF: right ventricular ejection fraction; SV: stroke volume.

| **Left ventricle** | **Bias** | **95 % LOA** | **ICC (95 % IC)** | **COV (%)** |
| --- | --- | --- | --- | --- |
| LV mass [g] | -13.91 | -61.2 to 33.4 | 0.932 (0.825 – 0.974) | 11.4 |
| LV EDV [ml] | 12.82 | -30.9 to 56.5 | 0.977 (0.939 – 0.991) | 10.4 |
| LV ESV [ml] | 8.22 | -31.9 to 48.3 | 0.972 (0.928 – 0.989) | 16.3 |
| LV SV [ml] | 4.42 | -22.1 to 31.0 | 0.921 (0.796 – 0.970) | 12.3 |
| LVEF [%] | -0.26 | -14.7 to 14.2 | 0.886 (0.704 – 0.956) | 17.0 |
| **Right ventricle** | **Bias** | **95 % LOA** | **ICC (95 % IC)** | **COV (%)** |
| RV EDV [ml] | -8.35 | -28.1 to 11.4 | 0.846 (0.600 – 0.941) | 6.8 |
| RV ESV [ml] | 2.99 | -28.0 to 34.0 | 0.968 (0.918 – 0.988) | 20.7 |
| RV SV [ml] | -9.41 | -48.7 to 29.9 | 0.688 (0.189 – 0.880) | 28.1 |
| RVEF [%] | -5.05 | -31.6 to 21.5 | 0.791 (0.457 – 0.919) | 25.6 |

**Table S6** Agreement between manual and automated uncorrected analyses in patients with low ejection fraction high gradient Aortic stenosis (n = 19). EDV: end-diastolic volume; ESV: end-systolic volume; LV: left ventricular; LVEF: left ventricular ejection fraction; RV: right ventricular; RVEF: right ventricular ejection fraction; SV: stroke volume.

| **Left ventricle** | **Bias** | **95 % LOA** | **ICC (95 % IC)** | **COV (%)** |
| --- | --- | --- | --- | --- |
| LV mass [g] | -12.58 | -61.7 to 36.5 | 0.934 (0.838 – 0.973) | 25.0 |
| LV EDV [ml] | 14.46 | -27.9 to 56.8 | 0.968 (0.921 – 0.987) | 10.2 |
| LV ESV [ml] | 10.33 | -25.4 to 46.0 | 0.975 (0.939 – 0.990) | 13.6 |
| LV SV [ml] | 3.91 | -22.1 to 29.9 | 0.882 (0.709 – 0.952) | 17.2 |
| LVEF [%] | -0.15 | -11.0 to 10.7 | 0.947 (0.868 – 0.978) | 14.3 |
| **Right ventricle** | **Bias** | **95 % LOA** | **ICC (95 % IC)** | **COV (%)** |
| RV EDV [ml] | -7.27 | -62.0 to 47.4 | 0.945 (0.865 – 0.978) | 17.5 |
| RV ESV [ml] | 4.86 | -39.8 to 49.5 | 0.945 (0.865 – 0.978) | 24.1 |
| RV SV [ml] | -8.87 | -48.9 to 31.2 | 0.724 (0.319 – 0.888) | 30.8 |
| RVEF [%] | -5.85 | -31.5 to 19.8 | 0.706 (0.275 – 0.881) | 31.0 |

**Table S7** Agreement between manual and automated uncorrected analyses in patients with low ejection fraction low gradient Aortic stenosis (n = 21). EDV: end-diastolic volume; ESV: end-systolic volume; LV: left ventricular; LVEF: left ventricular ejection fraction; RV: right ventricular; RVEF: right ventricular ejection fraction; SV: stroke volume.

| **Left ventricle** | **Bias** | **95 % LOA** | **ICC (95 % IC)** | **COV (%)** |
| --- | --- | --- | --- | --- |
| LV mass [g] | -10.65 | -42.9 to 21.6 | 0.965 (0.928 – 0.983) | 11.5 |
| LV EDV [ml] | 7.84 | -24,6 to 40.3 | 0.956 (0.909 – 0.979) | 13.3 |
| LV ESV [ml] | 2.78 | -19.5 to 25.1 | 0.952 (0.900 – 0.977) | 21.8 |
| LV SV [ml] | 5.10 | -19.5 to 29.7 | 0.931 (0.857 – 0.967) | 17.3 |
| LVEF [%] | -0.13 | -12.3 to 12.0 | 0.933 (0.861 – 0.968) | 10.4 |
| **Right ventricle** | **Bias** | **95 % LOA** | **ICC (95 % IC)** | **COV (%)** |
| RV EDV [ml] | -3.42 | -39.7 to 32.8 | 0.960 (0.916 – 0.981) | 14.1 |
| RV ESV [ml] | -2.11 | -28.9 to 24.7 | 0.940 (0.875 – 0.971) | 20.7 |
| RV SV [ml] | -1.29 | -30.0 to 27.5 | 0.911 (0.816 – 0.957) | 22.5 |
| RVEF [%] | -0.53 | -16.9 to 15.9 | 0.807 (0.599 – 0.907) | 16.6 |

**Table S8** Agreement between manual and automated uncorrected analyses in in patients with paradoxical low flow low gradient Aortic stenosis (n = 31). EDV: end-diastolic volume; ESV: end-systolic volume; LV: left ventricular; LVEF: left ventricular ejection fraction; RV: right ventricular; RVEF: right ventricular ejection fraction; SV: stroke volume.
